# Assessment of first-trimester utero-placental vascular morphology by 3D power Doppler ultrasound image analysis using a skeletonization algorithm: the Rotterdam Periconception Cohort

**DOI:** 10.1093/humrep/deac202

**Published:** 2022-09-19

**Authors:** Eline S de Vos, Anton H J Koning, Régine P M Steegers-Theunissen, Sten P Willemsen, Bas B van Rijn, Eric A P Steegers, Annemarie G M G J Mulders

**Affiliations:** Department of Obstetrics and Gynecology, Erasmus MC, University Medical Centre Rotterdam, Rotterdam, The Netherlands; Department of Pathology, Erasmus MC, University Medical Centre Rotterdam, Rotterdam, The Netherlands; Department of Obstetrics and Gynecology, Erasmus MC, University Medical Centre Rotterdam, Rotterdam, The Netherlands; Department of Obstetrics and Gynecology, Erasmus MC, University Medical Centre Rotterdam, Rotterdam, The Netherlands; Department of Biostatistics, Erasmus MC, University Medical Centre Rotterdam, Rotterdam, The Netherlands; Department of Obstetrics and Gynecology, Erasmus MC, University Medical Centre Rotterdam, Rotterdam, The Netherlands; Department of Obstetrics and Gynecology, Erasmus MC, University Medical Centre Rotterdam, Rotterdam, The Netherlands; Department of Obstetrics and Gynecology, Erasmus MC, University Medical Centre Rotterdam, Rotterdam, The Netherlands

**Keywords:** computer-assisted image processing, virtual reality, ultrasonography, placental vascular development, preeclampsia

## Abstract

**STUDY QUESTION:**

Can three-dimensional (3D) Power Doppler (PD) ultrasound and a skeletonization algorithm be used to assess first-trimester development of the utero-placental vascular morphology?

**SUMMARY ANSWER:**

The application of 3D PD ultrasonography and a skeletonization algorithm facilitates morphologic assessment of utero-placental vascular development in the first trimester and reveals less advanced vascular morphologic development in pregnancies with placenta-related complications than in pregnancies without placenta-related complications.

**WHAT IS KNOWN ALREADY:**

Suboptimal development of the utero-placental vasculature is one of the main contributors to the periconceptional origin of placenta-related complications. The nature and attribution of aberrant vascular structure and branching patterns remain unclear, as validated markers monitoring first-trimester utero-placental vascular morphologic development are lacking.

**STUDY DESIGN, SIZE, DURATION:**

In this prospective observational cohort, 214 ongoing pregnancies were included before 10 weeks gestational age (GA) at a tertiary hospital between January 2017 and July 2018, as a subcohort of the ongoing Rotterdam Periconception Cohort study.

**PARTICIPANTS/MATERIALS, SETTING, METHODS:**

By combining 3D PD ultrasonography and virtual reality, utero-placental vascular volume (uPVV) measurements were obtained at 7, 9 and 11 weeks GA. A skeletonization algorithm was applied to the uPVV measurements to generate the utero-placental vascular skeleton (uPVS), a network-like structure containing morphologic characteristics of the vasculature. Quantification of vascular morphology was performed by assigning a morphologic characteristic to each voxel in the uPVS (end-, vessel-, bifurcation- or crossing-point) and calculating total vascular network length. A Mann–Whitney *U* test was performed to investigate differences in morphologic development of the first-trimester utero-placental vasculature between pregnancies with and without placenta-related complications. Linear mixed models were used to estimate trajectories of the morphologic characteristics in the first trimester.

**MAIN RESULTS AND THE ROLE OF CHANCE:**

All morphologic characteristics of the utero-placental vasculature increased significantly in the first trimester (*P* < 0.005). In pregnancies with placenta-related complications (n = 54), utero-placental vascular branching was significantly less advanced at 9 weeks GA (vessel points *P* = 0.040, bifurcation points *P* = 0.050, crossing points *P* = 0.020, total network length *P* = 0.023). Morphologic growth trajectories remained similar after adjustment for parity, conception mode, foetal sex and occurrence of placenta-related complications.

**LIMITATIONS, REASONS FOR CAUTION:**

The tertiary setting of this prospective observational study provides high internal, but possibly limited external, validity. Extrapolation of the study’s findings should therefore be addressed with caution.

**WIDER IMPLICATIONS OF THE FINDINGS:**

The uPVS enables assessment of morphologic development of the first-trimester utero-placental vasculature. Further investigation of this innovative methodology needs to determine its added value for the assessment of (patho-) physiological utero-placental vascular development.

**STUDY FUNDING/COMPETING INTEREST(S):**

This research was funded by the Department of Obstetrics and Gynecology of the Erasmus MC, University Medical Centre, Rotterdam, The Netherlands. There are no conflicts of interest.

**TRIAL REGISTRATION NUMBER:**

Registered at the Dutch Trial Register (NTR6854).

## Introduction

The placenta is an organ vital for human reproduction that allows for exchange of nutrients, gasses and waste between the mother and foetus (Burton and Jauniaux, 2015). For optimal functioning, the placenta depends on successful development of the utero-placental vasculature (Brosens *et al.*, 2019a). Accordingly, deranged early utero-placental vascular development is one of the main causes of the major disorders of pregnancy, also known as placenta-related complications (Brosens *et al.*, 2011).

Critical steps in utero-placental vascular development occur before the 12th week of pregnancy (Degner *et al.*, 2017). Therefore, measures to prevent placenta-related complications are most effective when initiated in the preconception stage and in the first trimester, i.e. the periconception period (Steegers-Theunissen *et al.*, 2013; Poon *et al.*, 2018). Early identification of pregnancies at risk of these complications is one of the major challenges in present-day obstetric care (Meertens *et al.*, 2019a,b). Development of reliable screening methods for early identification of placenta-related complications is therefore of utmost importance. Non-invasive biomarkers for monitoring first-trimester utero-placental vascular development could be valuable tools to detect pregnancies at risk of placental disease. However, current strategies to develop these markers using either ultrasound techniques, or circulating biomarkers, or both, show inconsistent associations with placental function and clinical outcomes (Kuc *et al.*, 2011).

Our current understanding of the development of the utero-placental vasculature in humans is mostly based on histopathological examinations (Pijnenborg *et al.*, 1980; Starzyk *et al.*, 1997; Brosens *et al.*, 2019b; Parks and Catov, 2020). This approach is, of course, not suitable for *in vivo* assessment and longitudinal data collection, which requires non-invasive testing in ongoing pregnancies (Roberts *et al.*, 2017; Maric-Bilkan *et al.*, 2019). In the last decade, several study groups, including our own, have developed new non-invasive imaging methods for the *in vivo* investigation of utero-placental vascular development in early pregnancy (Mathewlynn and Collins, 2019; Exalto *et al.*, 2021). The application of offline image processing has been key in this research (Elsayes *et al.*, 2009; Collins *et al.*, 2012a,b; Roberts *et al.*, 2017; Stevenson *et al.*, 2018; Mathewlynn and Collins, 2019; Ridder *et al.*, 2019). Using Doppler techniques, ultrasonography allows for identification of the first-trimester placenta and most of its vascularization (Collins *et al.*, 2012a,b; Reus *et al.*, 2013). Offline image processing of three-dimensional (3D) power Doppler (PD) ultrasound enables semi-automated measurements of placental volume (PV) and utero-placental vascular volume (uPVV) (Collins *et al.*, 2012a,b; Reus *et al.*, 2013; Reijnders *et al.*, 2018). These techniques provide information on volumetric development of the placenta and utero-placental vasculature. However, the application of 3D PD ultrasound to asses vascular morphology, i.e. to describe the vascular structure and branching patterns of the utero-placental vasculature during pregnancy, has not yet been investigated (Espinoza *et al.*, 2006; Tongpob and Wyrwoll, 2021).

In this study, we aim to investigate morphologic development of the first-trimester human utero-placental vasculature *in vivo*. Using 3D PD ultrasound and advanced image processing, our objective is to generate the utero-placental vascular skeleton (uPVS), a structural representation of the utero-placental vascular anatomy. The uPVS provides information on the number and type of morphological features in the utero-placental vasculature. By combining the uPVS and volumetric measurements, we can calculate the density of vascular branching. Finally, using uPVS characteristics and density parameters, we will study morphologic development of the first-trimester utero-placental vasculature and investigate associations between morphologic variations and pregnancy outcome.

## Materials and methods

### Study design and ethical approval

We used data from the VIRTUAL Placenta study, which is embedded in the Rotterdam Periconception Cohort (MEC-2004-227), an ongoing observational study (Steegers-Theunissen *et al.*, 2016; Rousian *et al.*, 2021). The Virtual Placenta study was conducted in accordance with the ethical principles for medical research set out in the Declaration of Helsinki and was approved by the Institutional Review Board of the Erasmus Medical Centre on 2 June 2015 (MEC 2015-494). Women were eligible to participate if they had a minimum age of 18 years, carried a singleton pregnancy less than 10 weeks gestational age (GA) at inclusion and gave written informed consent. Both natural pregnancies and pregnancies achieved after IVF with or without ICSI were eligible for inclusion.

### Data collection

For all participants, at least two study visits were scheduled in the first trimester, at 7, 9 and 11 weeks GA.

For natural pregnancies in regular cycles, with a duration between 25 and 35 days, GA was calculated from the first day of last menstrual period (LMP). In case of unknown LMP or irregular cycles, GA was calculated from Crown-Rump-Length (CRL). If the two methods varied more than 6 days, the CRL-based GA was assumed the true GA. For fresh IVF/ICSI pregnancies, GA was calculated from oocyte retrieval day + 14 days. In case of cryopreserved embryo transfer, GA was calculated from transfer date + 19 days.

At enrolment, participants filled out a questionnaire on general characteristics and medical and obstetrical history. Height and weight measurements were standardized to calculate BMI at the first study visit.

Pregnancy outcomes were collected through a questionnaire and were complemented with official medical delivery records.

### Ultrasonography

Trained sonographers performed all ultrasounds using the GE Voluson E8 (GE, Zipf, Austria) with a transvaginal 6–12 MHz transducer. Standardized ultrasound settings were used and all participants were scanned according to protocol (quality: max; pulse repetition frequency (PRF): 0.6; wall motion filter (WMF): low1; compound resolution imaging (CRI): off; PD gain: −8.0) (Reijnders *et al.*, 2018).

All examinations comprised 2D CRL measurements and 3D sweeps of the whole gestational sac including the placenta. The utero-placental vasculature was visualized with a 3D PD sweep (Reijnders *et al.*, 2018). All ultrasound examinations were performed according to international guidelines on safe use of Doppler ultrasound in the first trimester of pregnancy (ALARA-principle) and were always <30 min to avoid unnecessary exposure (Drukker *et al.*, 2020).

### Offline 3D measurements for retrieving volumetric characteristics

Image quality was scored on a four-point scale ranging between zero (optimal) and three (unusable). The quality score was based on the presence of artefacts (as a result of embryonic or foetal movements causing blurring of the volume or acoustic shadowing), the ability to distinguish between myometrium and trophoblastic tissue, and completeness of the placenta (Reijnders *et al.*, 2018). Unusable images were excluded from the analysis.

The PV was measured using VOCAL software according to the previously published study protocol (Reijnders *et al.*, 2018). In short, the placental outline, identified based on differences in echogenicity between trophoblast and myometrium, was repeatedly traced in rotational steps of 15 degrees to calculate total pregnancy volume. Likewise, the gestational sac volume was calculated by tracing the gestational sac contours. The gestational sac volume was subtracted from the total pregnancy volume to calculate PV (cm^3^) (Reus *et al.*, 2013).

**Figure 1.**
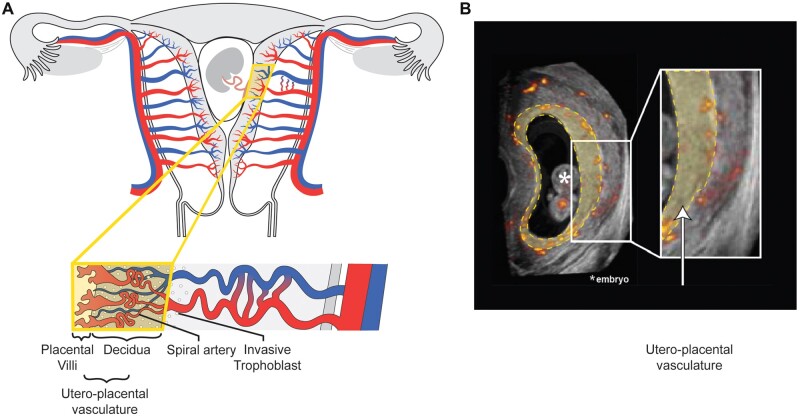
**Anatomy of utero-placental vasculature and corresponding virtual reality segmentation.** Overview of the utero-placental vasculature. (**A**) Schematic image of a pregnant uterus including the uterine and placental vasculature (based upon Burton *et al.*, 2009, red, arterial vasculature; blue, venous vasculature). In the magnified cross-section, the yellow box contains decidua, invasive extravillous trophoblast and placental tissue which form the interface of the utero-placental vasculature. The vascular structures within the utero-placental vasculature are confined to the maternal spiral arteries and their communicating anastomoses, and possibly vessels of the embryonic/foetal-placental blood space. (**B**) Two-dimensional (2D) power Doppler (PD) ultrasound image of a first-trimester pregnancy; all recorded vasculature is depicted in red-yellow colour. The yellow-highlighted section corresponds with the yellow box in the schematic image and forms the virtual reality (VR) segmentation. The white box contains a magnified cross-section. All vasculature in the VR segmentation is included in the utero-placental vascular skeleton (uPVS).

The uPVV was measured using a virtual reality (VR) desktop system with the V-Scope volume rendering application. The V-Scope application constructs a floating hologram from a 3D dataset, which can be viewed using the VR desktop system. The VR desktop system consists of a 3D-monitor, a pair of stereoscopic glasses that allow depth perception, a tracker to focus the holograms’ perspective and a 6-degrees-of-freedom mouse to manipulate the hologram in all three dimensions and perform measurements. Semi-automatic volume measurements of the utero-placental vasculature were obtained by thresholding the 8-bit (range 0–255) Doppler magnitude data. First, the lower threshold of PD signal intensity was set at a value of 100 for the most appropriate visualization of the vasculature in all scans (Reus *et al.*, 2014). As a result, only voxels with a PD signal intensity between 100 and 255 were included in subsequent vascular volume measurements. Next, PD artefacts, recognizable by their stripe-like appearance, were removed with a virtual eraser, i.e. deselecting voxels that were initially selected by the thresholding step. Then, by visually identifying the embryonic structures and differences in tissue echogenicity between uterus and placenta, VR segmentation was used to remove the grey-scale and PD signal within the embryo, the umbilical cord and the uterine tissue surrounding the placenta up to the margin of the placental-myometrial interface. This leaves PD signal only inside the region of interest. Lastly, the V-Scope application automatically calculated the volume of all voxels with PD signal intensity >100 within the VR segmentation to measure the uPVV (cm^3^), as published previously (Reijnders *et al.*, 2018). Following these offline image processing steps, the uPVV is defined as the vascular volume within the decidua, invasive extravillous trophoblast and placental tissue. Accordingly, the vascular structures in the uPVV are confined to (the distal ends of) the maternal spiral arteries and their communicating anastomoses, and possibly vessels of the embryonic/foetal-placental blood space (Fig. 1) (Burton *et al.*, 2009). At this stage, it is not possible with VR technology to make an absolute distinction between the maternal vasculature and embryonic/foetal blood space within the uPVV.

### Computer-assisted image processing for obtaining morphologic characteristics

Next, a skeletonization algorithm was applied to the uPVV segmentation to generate the uPVS. The skeletonization algorithm repeatedly peels off the outermost layer of voxels from the uPVV, reducing the diameter of the PD signal at each point in the vascular network until one central voxel remains, thereby creating a network-like structure representing the vascular morphology. The underlying theory of this technique was previously described and implemented in other disciplines, including other vascular datasets (Post *et al.*, 2016). To our knowledge, this application has never been used to post-process ultrasound data.

**Figure 2.**
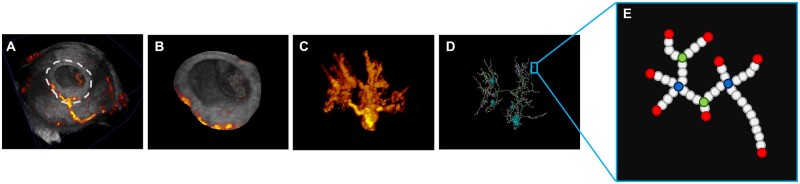
**Virtual reality segmentation and skeletonization of three-dimensional power Doppler ultrasound of a first-trimester pregnancy.** Panel **A** depicts a three-dimensional (3D) power Doppler (PD) ultrasound of a first-trimester pregnancy. Using virtual reality (VR) segmentation, myometrial tissue surrounding the trophoblast and embryonic structures in the gestational sac are erased. The VR segmentation of placental tissue is depicted in panel **B**. After removal of greyscale ultrasound signal, a 3D PD image of utero-placental vasculature (uPVV) remains, which is depicted in panel **C**. Then, a skeletonization algorithm is applied to the uPVV, resulting in the utero-placental vascular skeleton (uPVS), depicted in panel **D**. Panel **E** depicts a magnified portion of the uPVS. Each voxel is assigned a morphologic characteristic (red, endpoint; white, vessel point; green, bifurcation point; blue, crossing point). Three other characteristics are derived from the uPVS: I. Total network length, II. Average vessel length, III. Average vessel thickness.

Following the construction of the network, the skeletonization algorithm classifies each 26-connected voxel based on the number of neighbouring voxels as: (i) endpoint (1 neighbour), (ii) regular vessel point (2 neighbours), (iii) bifurcation point (3 neighbours) or (iv) crossing point (4 neighbours) (Fig. 2). We also observed clusters of accumulated voxels (≥5 neighbours) and single voxels (0 neighbours). Clusters result from the skeletonization of a ‘hollow’ PD signal, which the algorithm cannot successfully reduce to a single central voxel. Clusters of voxels and single voxels were regarded as PD noise or scanning artefacts and were excluded from analysis. Not all the voxels in the uPVS are necessarily connected to one central tree-like structure. Instead, the uPVS could also contain two or more distinct branches with no communicating anastomoses between them.

Summation of the total number of voxels in the uPVS, multiplied by the average distance in mm between the centres of two neighbouring voxels, resulted in (5) the total network length. Likewise, the average number of vessel points between two endpoints, bifurcation points or crossing points multiplied by the average voxel distance mentioned above, resulted in (6) the average vessel length. Last, the average number of voxels that were peeled off to reach the most central voxel in the skeletonization process was multiplied by the average voxel distance to calculate the average vascular thickness (7). Accordingly, the uPVS resulted in seven distinct morphologic characteristics of the utero-placental vasculature.

The ratio of these seven uPVS characteristics to the PV was calculated (uPVS:PV ratio) to determine the density of vascular branching within the placenta.

The ratio of the seven uPVS characteristics to the uPVV was also calculated (uPVS:uPVV ratio) to determine the density of vascular branching within the uPVV.

### Definitions of placenta-related complications

Placenta-related complications were specified as pregnancy-induced hypertension (PIH) or preeclampsia (PE) and/or foetal growth restriction (FGR), small-for-gestational age (SGA) and preterm birth (PTB). PIH was defined as a systolic blood pressure ≥140 mmHg and/or a diastolic blood pressure ≥90 mmHg after 20 weeks GA without signs of hypertension prior to pregnancy or presence of proteinuria (Croke, 2019). PE was defined as PIH with presence of more than 300 mg proteinuria in a 24-h period (Croke, 2019). FGR was defined as either a foetal abdominal circumference and/or estimated foetal weight (EFW) <10th percentile according to Hadlock curves, or a more than 20 percentile decrease on the growth curve, compared to previous measurements with a minimal timespan of two weeks between the measurements (Gordijn *et al.*, 2016). SGA was defined as birth weight <10th percentile on growth curves specific for GA at birth, parity and foetal sex and PTB was defined as a GA at birth below 37 weeks (259 days) (Zeve *et al.*, 2016; Vogel *et al.*, 2018).

### Statistical analysis

Study population characteristics were reported as mean with SD or percentage. Independent sample *t*-test and chi-square test were used to test for differences in population characteristics between pregnancies with and without placenta-related complications when appropriate. Morphologic characteristics of the utero-placental vasculature were reported as median with interquartile range (IQR). Spearman correlation analysis was performed to investigate cohesion between the individual characteristics within the uPVS and with PV and uPVV.

Principal component analysis (PCA) was applied to identify structural patterns in morphologic characteristics in the uPVS at 7, 9 and 11 weeks GA separately. PCA is a standard multivariate statistical technique that was used to aggregate morphologic characteristics in the uPVS based on the degree reciprocal correlation of the morphologic characteristics. All seven morphologic characteristics in the uPVS were entered in the PCA. We selected two principal components with the highest eigenvalues at 7, 9 and 11 weeks GA.

We applied a model for repeated measurements to the outcomes uPVS characteristics, uPVS/PV density and uPVS/uPVV density. Subsequently, we applied a maximum likelihood test to each model to test for differences at 7, 9 and 11 weeks GA. Mann–Whitney *U* test was performed to test for differences in the uPVS, uPVS/PV density and uPVS/uPVV density between pregnancies with and without placenta-related complications at 7, 9 and 11 weeks GA. Linear mixed models were used to estimate trajectories of the uPVS characteristics using a quadratic relation with GA and a random intercept per pregnancy. The effect of conception mode, parity, foetal sex and occurrence of placenta-related complication on trajectories of uPVS characteristics was investigated by entering corresponding interaction terms to the model.

All analyses were performed using SPSS software (version 25.0; SPSS Inc., Chicago, IL, USA) and R (version 3.5.0, R Core Team, Vienna, Austria, 2018). *P*-values ≤0.05 were considered statistically significant.

## Results

### Study population

**Figure 3.**
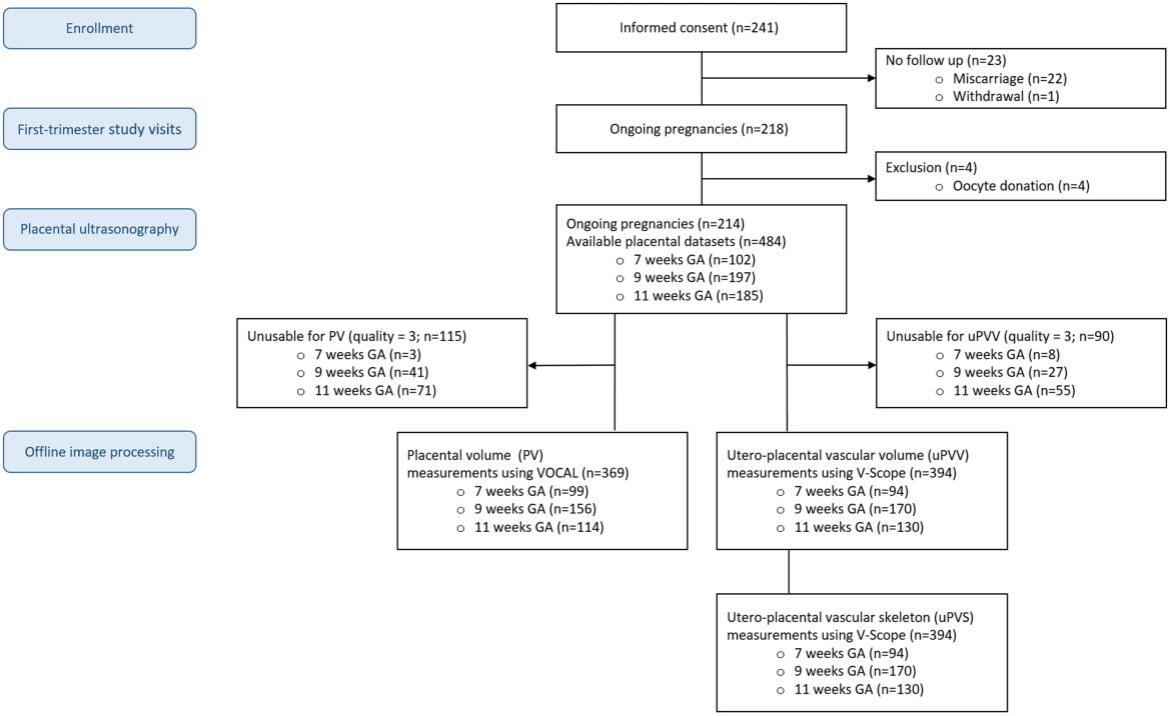
**Flowchart of participant selection.** GA, gestational age.

For the present study, 214 women of the VIRTUAL placenta cohort were eligible for inclusion. Figure 3 depicts the flowchart of participant selection. Overall, 81% of all ultrasound data was of sufficient quality to perform uPVV and subsequently uPVS measurements (Fig. 3). The most frequent reasons for classifying an ultrasound dataset as unusable were shadowing (40.0%) or the inability to capture the whole placenta in the 3D PD sweep (54.4%). The remaining 5.6% was classified as unusable due to errors in ultrasound settings or movement artefacts (data not shown). Table I depicts patient characteristics of the study population at baseline. There were 122 women (57.0%) who were nulliparous and 87 women (40.7%) conceived via IVF/ICSI. A total of 54 (25.2%) pregnancies developed one or more placenta-related complications (PIH, PE and/or FGR, SGA and PTB). At baseline, there were no differences in patient characteristics between women who did and did not develop placenta-related complications.

**Table I:** Characteristics of study population in total cohort and in pregnancies with and without placenta-related complications.

Characteristic	Total cohort (n = 214) Mean (SD); n (%)	Without (n = 160) Mean (SD); n (%)	With (n = 54) Mean (SD); n (%)	*P*-value
**Maternal characteristics**				
Age, years	32.3 (4.5)	32.6 (4.5)	31.4 (4.3)	0.094
Nulliparous	122 (57.0%)	92 (57.5%)	30 (55.6%)	0.803
IVF/ICSI	87 (40.7%)	71 (44.4%)	16 (29.6%)	0.056
Geographic origin				0.481
Dutch	166 (77.6%)	128 (80.0%)	38 (70.4%)	
Western	6 (2.8%)	5 (3.1%)	1 (1.9%)	
Non-Western	38 (17.8%)	26 (16.3%)	12 (22.2%)	
Educational level				0.196
Low	18 (8.4%)	11 (6.9%)	7 (13.0%)	
Intermediate	69 (32.2%)	56 (35.0%)	13 (24.1%)	
High	123 (57.5%)	92 (57.5%)	31 (57.4%)	
BMI first visit, (kg/m^2^)	25.9 (5.2)	25.6 (5.00)	26.8 (5.7)	0.155
Folic acid supplementation, yes	178 (83.6%)	138 (86.3%)	40 (74.1)	0.066
Alcohol consumption, yes	57 (26.6%)	47 (29.4%)	10 (18.5)	0.119
Smoking, yes	28 (13.1%)	19 (11.9%)	9 (16.7%)	0.367
**Neonatal outcomes**				
Foetal sex, boys	106 (49.5%)	76 (47.5%)	30 (55.6%)	0.433
GA at birth, days	267 (30)	271 (29)	258 (30)	**0.001**
Birth weight, g	3177 (690)	3385 (589)	2576 (608)	**<0.001**
Hoftiezer percentiles	46 (29)	53 (26)	26 (28)	**<0.001**
Congenital anomalities	8 (3.7%)	8 (5.0%)	0 (0.0%)	0.094
**Placenta-related complications^a^**	54 (25.2%)	0 (0.0%)	15 (27.8%)	**<0.001**
PIH^b^	9 (4.2%)	0 (0.0%)	9 (16.9%)	**<0.001**
PE^b^	7 (3.3%)	0 (0.0%	7 (13.0%)	**<0.001**
FGR^b^	15 (7.0%)	0 (0.0%)	9 (16.7%)	**<0.001**
SGA^b^	19 (8.9%)	0 (0.0%)	7 (13.0%)	**<0.001**
PTB^b^	23 (10.7%)	0 (0.0%)	23 (42.6%)	**<0.001**

a Placenta-related complications are specified as PIH, PE and/or FGR/SGA and PTB.

^b^ Diagnoses may be overlapping. FGR, foetal growth restriction; PE, preeclampsia; PIH, pregnancy-induced hypertension; SGA, small-for-gestational age; PTB, preterm birth.

Significance at *P*-value ≤ 0.05 (depicted in bold) assessed with independent sample t-test and chi-square test when appropriate.

### Utero-placental vascular morphology

**Figure 4.**
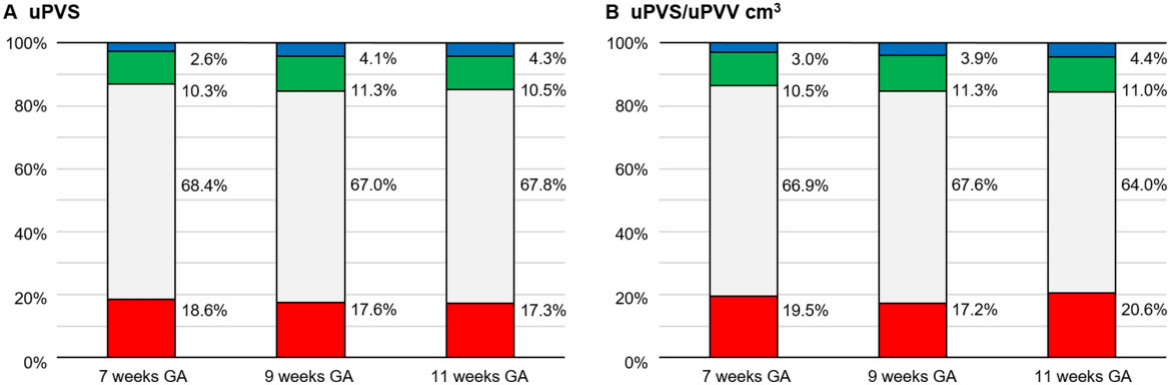
**Bar chart of individual morphologic characteristics in the utero-placental vascular skeleton (uPVS).** Bar chart of the distribution of individual morphologic characteristics in the utero-placental vascular skeleton (uPVS) (**A**), and per cm^3^ utero-placental vascular volume (uPVV) (**B**), showing the proportional number of endpoints, vessel points, bifurcation points and crossing points in the uPVS at 7, 9, an 11 weeks gestational age (GA). Red, endpoint; white, vessel point; green, bifurcation point; blue, crossing point.

The distribution of endpoints, vessel points, bifurcation points and crossing points in the uPVS and the distribution of uPVS characteristics per cm^3^ uPVV is depicted in the Fig. 4. This figure summarizes the morphology of the utero-placental vasculature at 7, 9 and 11 weeks GA and shows consistent morphologic patterns throughout the first trimester. Approximately two-thirds of the uPVS comprised vessel points. Almost one-fifth of the uPVS is endpoint and the remaining 12–15% consists of branches, which are either bifurcation points (∼75%) or crossing points (∼25%).

### Correlation between PV, uPVV and uPVS

**Figure 5.**
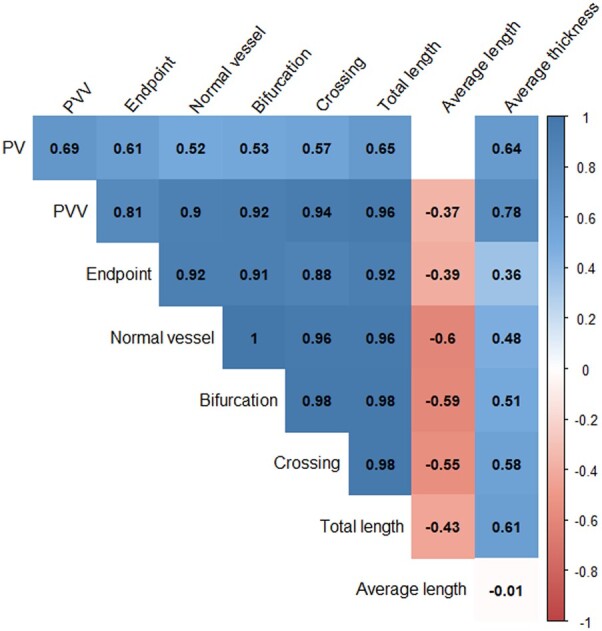
**Correlation plot of placental volume (PV), utero-placental vascular volume (uPVV) and utero-placental vascular skeleton (uPVS) characteristics.** Relationship between placental volume (PV), utero-placental vascular volume (uPVV) and utero-placental vascular skeleton (uPVS). Coloured squares represent significant correlations. Blue and red squares represent positive and negative correlations, respectively. A higher colour saturation indicates a stronger correlation. White squares represent absence of a significant correlation. Spearman correlation analysis is used. All *P*-values <0.001. No adjustments are made in this model.

Figure 5 depicts moderate to strong positive correlations between uPVS characteristics and PV (end-: *ρ* = 0.61, vessel-: *ρ* = 0.52, bifurcation-: *ρ* = 0.53, crossing-points: *ρ* = 0.57, total length: *ρ* = 0.65, average thickness: *ρ* = 0.64) and strong positive correlations between uPVS characteristics and uPVV (end-: *ρ* = 0.81, vessel-: *ρ* = 0.90, bifurcation-: *ρ* = 0.92, crossing-points: *ρ* = 0.94, total length: *ρ* = 0.96, average thickness: *ρ* = 0.78). All noted spearman correlations were statistically significant with *P*-values <0.001. In addition, all uPVS characteristics were interchangeably strongly and positively correlated with one another (range *ρ* = 0.88–1.00, *P* < 0.001), except for average length, which was negatively correlated with other uPVS characteristics (range *ρ* = −0.39; −0.60, *P* ≤ 0.001; 0.949). A low to moderate positive correlation was found between the characteristics of the uPVS and average thickness (range *ρ* = −0.01; 0.61, *P* ≤ 0.001; 0.949).

### PCA of uPVS characteristics

**Figure 6.**
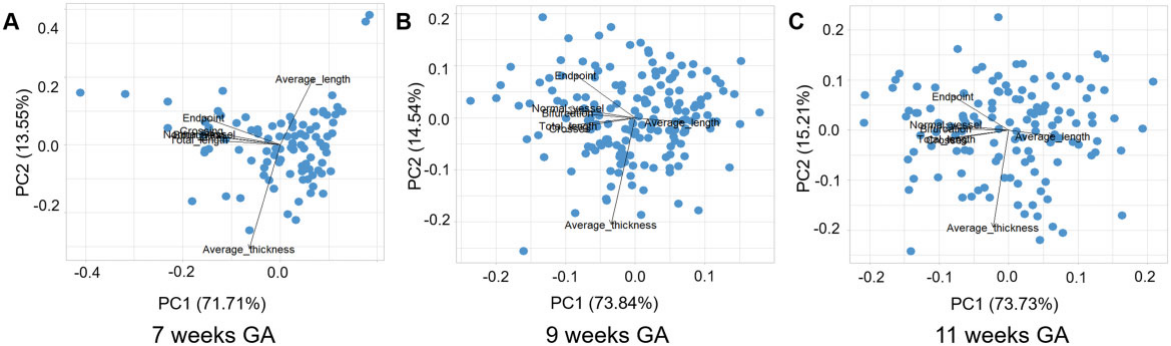
**Principal component analysis of individual utero-placental vascular skeleton (uPVS) characteristics projected onto the first two principal components.** Blue dots represent the individual observations in the dataset. (**A**) Principal component analysis (PCA) plot of the utero-placental vascular skeleton (uPVS) at 7 weeks gestational age (GA) (n = 94). (**B**) PCA plot of uPVS at 9 weeks GA (n = 170). (**C**) PCA plot of uPVS at 11 weeks GA (n = 129). PC1, principal component 1; PC2, principal component 2.

Figure 6 depicts the PCA plot of the uPVS characteristics at 7, 9 and 11 weeks GA. The first principal component (PC1) was associated with a high number of uPVS endpoints, vessel points, bifurcation points, crossing points and total length and explained 71.71–73.84–73.73% of total variance of uPVS at 7–9–11 weeks GA, respectively. The second principal component (PC2) was associated with high vascular thickness and explained 13.55–14.54–15.21% of total variance at 7–9–11 weeks GA, respectively. Together, the uPVS patterns reflected by PC1 and PC2 explained 85.3% at 7 weeks GA, 88.4% at 9 weeks GA and 88.9% at 11 weeks GA (Fig. 6).

### uPVS development and density of vascular branching in the first trimester

**Figure 7.**
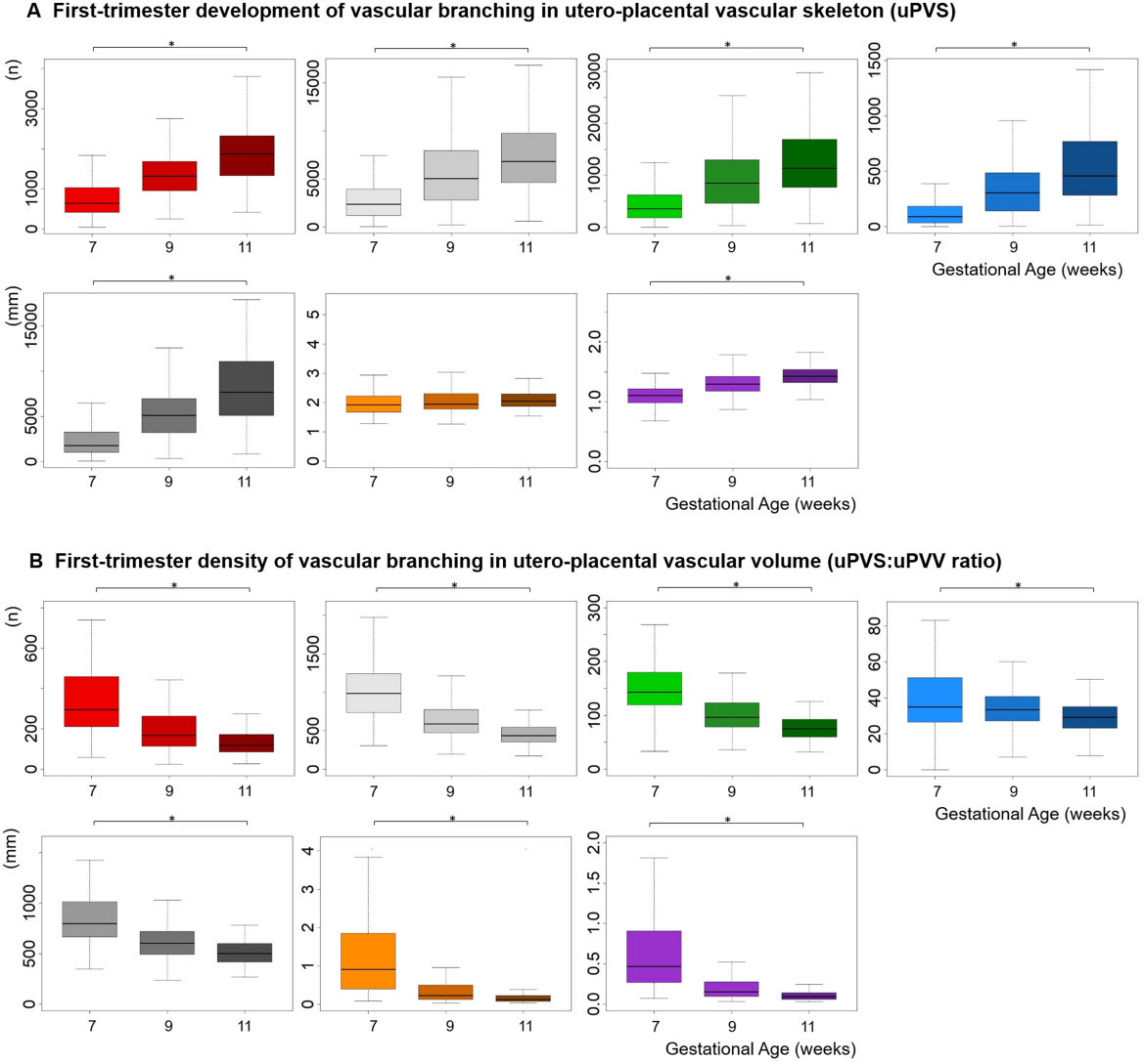
**First-trimester development of utero-placental vascular skeleton (uPVS).** Development of (**A**) individual uPVS characteristics, and (**B**) uPVS:uPVV ratio in the first trimester at 7 (n = 94), 9 (n = 170) and 11 (n = 129) weeks GA. Each uPVS characteristic is depicted by a separate colour. Red, endpoint; white, vessel point; green, bifurcation point; blue, crossing point; grey, total length; orange, average length; purple, average thickness. *Significance at *P*≤0.05 assessed with ANOVA. uPVS, utero-placental vascular skeleton; uPVV, utero-placental vascular volume; GA, gestational age.

All uPVS characteristics increased significantly throughout the first trimester, except for uPVS average length, which remained constant between 7, 9 and 11 weeks GA (Fig. 7A). Both PV and uPVV increase significantly throughout the first trimester (Reijnders *et al.*, 2021). The PV and the uPVV increased at a higher rate than the amounts of uPVS characteristics, resulting in a significant decrease in the density of vascular branching in PV (uPVS:PV ratio) (data not shown) and uPVV (uPVS:uPVV ratio) between 7–9–11 weeks GA (Fig. 7B).

Additionally, there was a positive slope of the trajectories (n = 189) of all uPVS characteristics in the first trimester. The slopes were not significantly different between subgroups defined by parity, conception mode, foetal sex or occurrence of placenta-related complications (data not shown).

### Development of the uPVS and density of vascular branching in pregnancies with and without placenta-related complications

**Figure 8.**
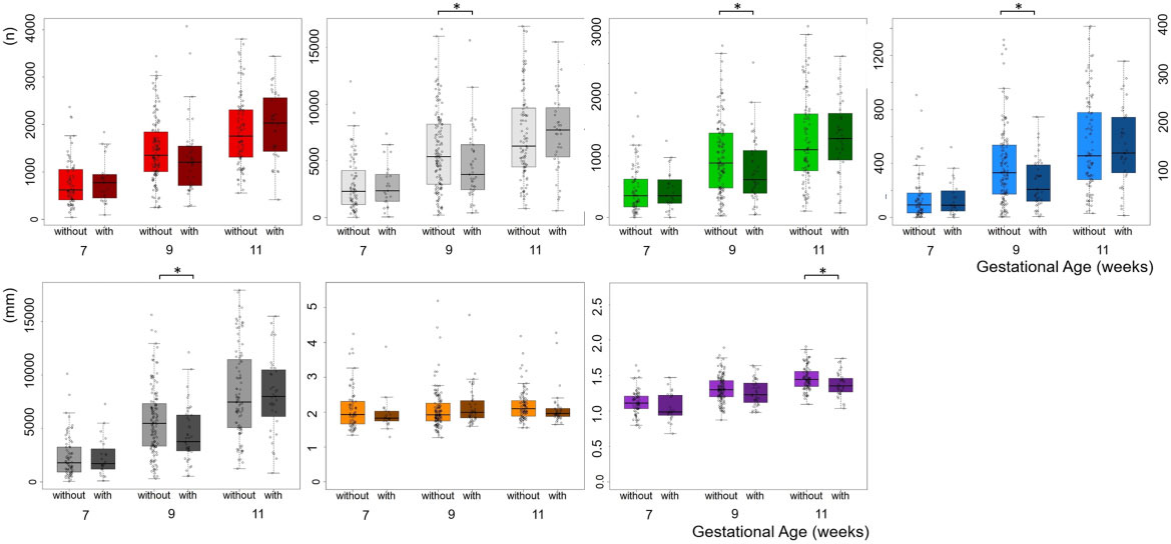
**First-trimester *in vivo* morphologic development of utero-placental vasculature in pregnancies with and without placenta-related complications, assessed with the utero-placental vascular skeleton (uPVS).** Differences in first-trimester uPVS development (n or mm) at 7, 9 and 11 weeks GA between pregnancies with and without placenta-related complications. Placenta-related complications are specified as PIH or PE and/or FGR, PTB and SGA; diagnoses may be overlapping. Each uPVS characteristic is depicted by a separate colour. Pregnancies with a placenta-related complication are depicted in a darker shade. Red, endpoint; white, vessel point; green, bifurcation point; blue, crossing point; grey, total length; orange, average length; purple, average thickness. uPVS, utero-placental vascular skeleton; GA, gestational age; IQR, interquartile range; PIH, pregnancy-induced hypertension; PE, preeclampsia; FGR, foetal growth restriction; PTB, pre-term birth; SGA, small-for-gestational age. *Significance at *P*≤0.05 assessed with Mann–Whitney *U* test.

After stratification for the occurrence of placenta-related complications, we observed no significant differences in the uPVS characteristics between pregnancies with and without placenta-related complications at 7 weeks GA (n = 94) (Fig. 8). At 9 weeks GA (n = 170), the uPVS in pregnancies with placenta-related complications had significantly fewer number of vessel points (*P* = 0.040), bifurcation points (*P* = 0.050) and crossing points (*P* = 0.020) and a shorter total network length (*P* = 0.023) (Fig. 8). At 11 weeks GA (n = 129), uPVS average vascular thickness was significantly lower in pregnancies with placenta-related complications (*P* = 0.007) (Fig. 8). For the other uPVS characteristics, no significant differences were observed between the two groups at 11 weeks GA.

After stratification for the occurrence of placenta-related complications, the density of vascular branching in PV showed no differences at 7 and 9 weeks GA. However, at 11 weeks GA, pregnancies with placenta-related complications showed an increased density of vascular branching in PV for the uPVS characteristics: end-, vessel and bifurcation points (data not shown). In addition, pregnancies with a placenta-related complication showed increased density of vascular branching in the uPVV, reflected by an increase in uPVS vessel points: uPVV ratio in at 7 weeks gestation (*P* = 0.047) (Table II). At 7 and 9 weeks GA, pregnancies with placenta-related complications also showed an increased density of vascular branching in the uPVV for the other morphologic characteristics, but the difference from pregnancies without placenta-related complications was not statistically significant. At 11 weeks GA, pregnancies with placenta-related complications showed an increased density of vascular branching in the uPVV, reflected by an increase in uPVS:uPVV ratio for vessel points (*P* = 0.005), bifurcation points (*P* = 0.002), crossing points (*P* = 0.007) and total length (*P* = 0.022).

**Table II:** First-trimester density of vascular branching in the utero-placental vascular volume (uPVS:uPVV ratio) in pregnancies with and without placenta-related complications.

Morphologic characteristics	7 weeks GA			9 weeks GA			11 weeks GA		
	without	with		without	with		Without	with	
	(n = 71)	(n = 23)		(n = 129)	(n = 41)		(n = 94)	(n = 35)	
	*Median (IQR)*	*Median (IQR)*	*P*-value	*Median (IQR)*	*Median (IQR)*	*P*-value	*Median (IQR)*	*Median (IQR)*	*P*-value
uPVS endpoints:uPVV ratio (n/cm^3^)	289.6 (201.1; 405.1)	368.9 (221.4; 536.8)	0.138	163.5 (105.3; 240.5)	206.1 (120.2; 319.8)	0.079	113.0 (80.6; 158.0)	149.7 (91.0; 200.7)	0.061
uPVS vessel points:uPVV ratio (n/cm^3^)	940.8 (731.2; 1172.0)	1108.9 (848.4; 1452.8)	**0.047**	586.7 (469.1; 750.7)	645.7 (487.7; 852.5)	0.148	423.2 (335.5; 506.6)	514.3 (381.7; 623.5)	**0.005**
uPVS bifurcation points:uPVV ratio (n/cm^3^)	135.9 (111.2; 169.7)	166.0 (126.2; 227.8)	0.072	94.7 (77.9; 121.1)	109.6 (83.3; 131.7)	0.125	72.9 (56.2; 84.1)	84.9 (67.1; 103.4)	**0.002**
uPVS crossing points:uPVV ratio (n/cm^3^)	33.3 (24.5; 50.0)	44.9 (29.0; 55.0)	0.070	33.6 (26.9; 41.6)	27.6 (31.7; 41.3)	0.725	28.4 (22.9; 34.2)	27.9 (33.7; 37.5)	**0.007**
uPVS total length:uPVV ratio (mm/cm^3^)	778.1 (663.0; 952.6)	936.8 (660.0; 1196.0)	0.090	591.0 (488.1; 707.9)	651.6 (515.0; 856.7)	0.091	492.9 (399.0; 573.1)	467.6 (558.1; 656.7)	**0.022**
uPVS average length:uPVV ratio (mm/cm^3^)	0.9 (0.4; 2.2)	0.9 (0.4; 1.4)	0.829	0.20 (0.1; 0.5)	0.3 (0.2; 0.6)	**0.020**	0.1 (0.1; 0.2)	0.1 (0.1; 0.2)	0.619
uPVS average thickness:uPVV ratio (mm/cm^3^)	0.5 (0.3; 1.1)	0.6 (0.3; 0.7)	0.802	0.1 (0.1; 0.3)	0.2 (0.1; 0.3)	**0.017**	0.1 (0.1; 0.2)	0.1 (0.1; 0.1)	0.567

Differences in first-trimester uPVS:uPVV ratio development (n/cm^3^ or mm/cm^3^) at 7, 9 and 11 weeks GA between pregnancies with and without placenta-related complications. Placenta-related complications are specified as PIH, PE and/or FGR/SGA and PTB; diagnoses may be overlapping. uPVS:uPVV ratio, utero-placental vascular skeleton:utero-placental vascular volume ratio; GA, gestational age; IQR, interquartile range; PIH, pregnancy-induced hypertension; PE, preeclampsia; FGR, foetal growth restriction; SGA, small-for-gestational age; PTB, pre-term birth. Significance at *P* ≤ 0.05 (depicted in bold) assessed with Mann–Whitney *U* test.

## Discussion

In this study, we combined first-trimester longitudinal 3D PD ultrasound, VR and a skeletonization algorithm to generate the uPVS as a morphologic representation of the utero-placental vascular network comprised seven distinct morphologic characteristics. We observed that the morphologic pattern of the first-trimester utero-placental vasculature, reflected by the distribution of uPVS characteristics, remains constant throughout the first trimester. We also observed that the length and number of branches in the first-trimester utero-placental vasculature increased significantly with advancing GA, as reflected by an increase in all characteristics of the uPVS between 7–9–11 weeks GA. The very strong correlations we observed between the uPVS characteristics and the uPVV, imply that the morphologic features measured by the uPVS are indeed a reflection of the utero-placental vasculature. It is therefore our presumption that the uPVS, as an imaging marker of vascular morphology, can be used to assess morphologic development and growth of the utero-placental vasculature in the first trimester.

Following the methodology of the uPVV measurements, the VR segmentation used to construct the uPVS includes the PD signal in placental tissue from the outlining of the gestational sac up to the placental–myometrial interface. In the first-trimester, this tissue segment mostly exists of decidua and extravillous trophoblast and the amount of chorionic villi is limited (Jauniaux *et al.*, 1991). From the fifth week onwards, embryonic capillaries arise in the chorionic villi (Burton *et al.*, 2009; van Oppenraaij *et al.*, 2009). Histopathologic investigation of first-trimester chorionic villi has shown the percentage of villous stroma area occupied by vascular elements covers only 0.7% by 5 weeks GA and only increases up to 2.5% at 10 weeks GA (te Velde *et al.*, 1997). Although the embryonic capillaries in the villi are connected with the umbilical cord and embryonic heart at 6 weeks GA, flow in the chorionic capillaries is not established until there is the presence of enucleated embryonic erythrocytes, which are produced from 8 weeks GA onwards (Burton *et al.*, 2009). Given the small contribution of embryonic vascular structures to the VR segmentation and the restricted presence of embryonic flow in the first trimester, we assume that the contribution of embryonic blood space in the uPVS is limited. Therefore we consider that the uPVS mainly reflects morphologic development of the maternal spiral arteries.

Previously, histopathological studies have described how the spiral arteries terminate in a capillary plexus just beneath the uterine epithelium (Burton *et al.*, 2009). Using the uPVS to describe vascular morphology, we observed a network-like structure with many communications in the first-trimester utero-placental vasculature. Combining 3D PD ultrasound and a skeletonization algorithm, we were able to verify the presence of these spiral artery anastomoses in the decidua of humans *in vivo*.

The uPVS demonstrates differences in first-trimester utero-placental vascular morphologic development between pregnancies with and without placenta-related complications, specified as PIH, PE and/or FGR, SGA and PTB. In pregnancies with placenta-related complications, utero-placental vascular branching, as reflected by the number of each of the uPVS characteristics, is reduced throughout the first trimester. Further, in pregnancies with placenta-related complications, utero-placental vascular calibre, as measured by uPVS average thickness, was revealed to be significantly smaller at 11 weeks GA. In addition, at 11 weeks GA, the density of vascular branching in uPVV is significantly higher, which reflects altered vascular development. These results are in line with our current, albeit limited, knowledge on morphologic development of the utero-placental vasculature. For example, research of histopathological examinations in term pregnancies (Brosens *et al.*, 1972; Labarrere *et al.*, 2017), has shown differences in lumen diameter, wall thickness, arterial density and vascular tortuosity in basal and spiral arteries in preeclamptic women compared to women with uncomplicated pregnancies (Starzyk *et al.*, 1997). Similarly, reduced vessel lumen was found in term placentas of pregnancies complicated by FGR (Junaid *et al.*, 2014). Using the uPVS, these differences in utero-placental vascular development between women with and without placenta-related complications can already be observed between 7 and 11 weeks of GA. Our observations comply with generally accepted paradigm that the first trimester is a critical time in utero-placental vascular development (Sato, 2020).

In normal pregnancies, trophoblast plugging of the spiral arteries starts around 7 weeks GA, restricting flow of oxygenated maternal blood to the placenta and provoking hemodynamic changes in the upstream uterine vasculature (Roberts *et al.*, 2017; James *et al.*, 2018; Knofler *et al.*, 2019). Premature disintegration of trophoblast plugs induces heightened blood flow into the intervillous space, causing ischemia–reperfusion injury and placental oxidative stress. Aberrant formation and resolving of endovascular trophoblast plugs prohibits adequate vascular remodelling (Staff *et al.*, 2020) and is associated with placenta-related complications (Burton and Jauniaux, 2017; Allerkamp *et al.*, 2021). In our study, premature unplugging of the spiral arteries might be reflected by both reduced vascular branching and thickness in pregnancies with placenta-related complications.

Recent evidence suggests that beside the spiral arteries, the maternal radial and arcuate arteries also undergo substantial physiological vascular remodelling to facilitate a healthy pregnancy outcome (Rennie *et al.*, 2016; Roberts *et al.*, 2017; Allerkamp *et al.*, 2021). Conforming to the methodology of the VR segmentation, the PD signal from the arcuate, radial and proximal segments of the spiral arteries, which extend through the myometrial tissue of the uterus, is not included in the uPVS. Therefore, we were not able to study the morphologic development of the radial and arcuate arteries in this study. For future research, we recommend to include the radial and arcuate arteries when investigating morphologic development of the utero-placental circulation.

Our study is unique due to its original focus on the development of a new methodology to assess morphologic development of the first-trimester human utero-placental vasculature *in vivo*. The main strengths of our study are longitudinal high quality data collection of 3D PD ultrasound from early first trimester and the use of validated uPVV measurements for generation of the uPVS. Furthermore, participants were included from a single hospital cohort using standardized protocols for ultrasonography, and well-trained researchers performed all offline measurements. Also, by applying VR segmentation, we are able to use the depth dimension of the 3D PD data to its fullest potential, which increases the accuracy of our measurements.

Our study has a few limitations. All participants were recruited from a tertiary referral hospital. As such, the study population mainly consists of high-risk pregnancies and over 40% conceived via IVF/ICSI. This design implies high internal, but limited external, validity. Therefore, extrapolating of these findings to a general population requires additional investigation. As result of our standardized settings and threshold value, we were not able to account for the influence of subject-specific characteristics such as BMI on tissue attenuation. Tissue attenuation could reduce the comparability of vascular measurements between participants and should always be considered when performing ultrasound measurements. However, we argue that structural measurements such as the uPVS, when compared to volumetric or flow measurements, are likely less impacted by tissue attenuation. Recently, the group of Collins and colleagues has proposed strategies to minimize the impact of differences in tissue attenuation on vascular measurements by using individual sub-noise gain levels during ultrasound acquisition or by using fractional moving blood volume during image processing (Collins et al., 2012c, 2017). For future data collection, we will adjust our ultrasound acquisition protocol by incorporating PD sub-noise gain settings to account for subject variability.

Despite our aim to schedule three study visits for each subject in the first trimester, we were not able to obtain usable 3D PD ultrasounds at 7, 9 and 11 weeks GA for each participant. As a result, the number of uPVS datasets at 7 and 11 weeks GA was substantially lower than at 9 weeks GA. Insufficient power might explain why we observed no statistically significant differences in utero-placental vascular morphology at 7 and 11 weeks GA between pregnancies with and without placenta-related complications.

Lastly, the group of participants with placenta-related complications is highly heterogeneous, comprising of PIH, PE and/or FGR, SGA and PTB. These complications share etiologic similarities involving aberrant development of the utero-placental vasculature. However, their pathophysiology is not identical. Therefore, the associations might be more profound if complications are analysed separately. As this study was not designed for this purpose, it has not enough power to perform these secondary analyses.

## Conclusion

To our knowledge, this is the first study to investigate morphologic development of the human utero-placental vasculature in the first trimester of pregnancy *in vivo*. Using 3D PD ultrasound and computer-assisted image processing, we generated the uPVS as an imaging marker for morphologic development of the utero-placental vasculature. Importantly, we observed differences in utero-placental vascular morphology between pregnancies with and without placenta-related complications. Therefore, this study justifies further research focusing on vascular morphology for the evaluation of (patho-) physiological utero-placental vascular development in pregnancy.

## Data Availability

The data underlying this article will be shared on reasonable request to the corresponding author.
